# Medical Department of the St. Louis University

**Published:** 1844-03

**Authors:** 


MEDICAL DEPARTMENT OF THE ST. LOUIS UNIVERSITY.

The newspapers of St. Louis report an unfortunate occurrence, as having taken place in regard to this school. It seems that a vault, containing subjects for dissection, was broken open, and a body partly dissected taken out, and left exposed upon the commons. A mob was the consequence: the college building was attacked; the windows were broken, and the museum pertaining to the school destroyed. The latter is represented as a great loss, many of the articles having been obtained from Europe at considerable expense, and others, prepared in this country, having required much time and labor in their preparation. All, it is stated by the newspapers, are destroyed or carried way by the mob. This is an occurrence deeply to be deplored. Such movements are contagious. They who have the supervision of anatomical affairs in schools of medicine cannot be too careful to avoid every cause which might provoke a similar outbreak of popular vengeance.

Since writing the above, we have received a paper containing a notice of Professor Linton's valedictory address, delivered to the graduates of the institution, eleven in number, on the last evening of February. No allusion is made in the address to the recent losses, but promises are made of large additions to their means of instruction before another season. The commencement was announced in the papers of the city, and went off quietly, the populace having, apparently, been appeased by seeing interred in the city cemetery the *debris* of the dissecting tables and anatomical museum. Y.

